# Electronic Health Record Time Allocation Among Primary Care Clinicians at the Veterans Health Administration Using Virtual Observations

**DOI:** 10.1007/s11606-024-09328-y

**Published:** 2025-01-06

**Authors:** Stefanie A. Deeds, Scott L. Hagan, Sara E. Kath, Leslie L. Taylor, Ashok S. Reddy, Karin M. Nelson

**Affiliations:** 1 VA Puget Sound Health Care System, Seattle, WA USA; 2 Division of General Internal Medicine, University of Washington, Seattle, WA USA

**Keywords:** Electronic health records, Veterans, Primary care

## Abstract

**Background:**

Prior research has shown that primary care clinicians (PCPs) spend a large portion of clinic visits on tasks within the electronic health record (EHR). However, no time allocation studies have been done in the Veterans Health Administration (VHA) and little is known about EHR time spent during virtual visits.

**Objective:**

To estimate the proportion of clinician time spent working within the EHR during primary care visits at VHA clinics.

**Design:**

We used a time study software (WorkStudy+) adapted from prior research to collect time-motion data via remote observation of in-person and virtual visits.

**Participants:**

23 PCPs (including physicians and nurse practitioners) from 3 regional VA sites.

**Main Measures:**

Proportion of observed time spent interfacing with the EHR during a primary care visit.

**Key Results:**

Of 211 primary care visits observed, the average visit length was 23.9 min in-person, 21.2 min by phone, and 29.3 min on video. The percentage of time spent on EHR work during the visit was 35% for in-person visits, 46% for phone visits, and 39% for video visits. During *n*=39 4-h clinic sessions, PCPs spent 54 min completing administrative tasks between patient visits, with 44% of time spent on documentation, 14% on chart review, and 14% on placing orders.

**Conclusions:**

PCPs at the VHA spend between one-third and one-half of each patient visit interfacing with the EHR. Most of this time is spent on documentation and chart review. Less time was spent in the EHR during in-person visits compared to virtual visits, suggesting that clinicians limit EHR task completion when the patient is present. Between patient visits during clinic sessions, PCPs spend 75% of their time working in the EHR. In total, this represents over 2 h per half-day clinic session spent on EHR tasks.

**Supplementary Information:**

The online version contains supplementary material available at 10.1007/s11606-024-09328-y.

## INTRODUCTION

The use of electronic health records (EHRs) in healthcare has increased the administrative burden for primary care clinicians. Clinicians spend more than half their time involved in administrative work related to patient care, including documentation, order entry, and chart review.^1–3^ Prior work has demonstrated that the large administrative burden of EHRs has directly contributed to clinician burnout.^4–6^ Moreover, the use of EHRs can have a direct impact on patient care, as clinicians using the EHR may employ less patient-centered communication compared to those using a paper chart.^7^

The Veterans Health Administration (VHA), overseeing one of the largest integrated healthcare system in the USA, is committed to reducing the administrative burdens faced by clinicians, particularly as it relates to its EHR, the Computerized Patient Records System (CPRS).^8,9^ Although there is increasing concern among primary care clinicians (PCPs) about the time burden from electronic administrative work,^10^ there is limited information on how VHA PCPs spend their time interacting with the VHA’s EHR and what impact the EHR may have on overall clinical workload. Additionally, although primary care visits have shifted to provide more video and phone care,^11^ we have limited information on the use of the EHR during video and phone visits.

Direct observational methods provide a more reliable measure of time spent on activities than self-report, and prior time allocation studies have developed methods of direct observation to track time spent using the EHR among healthcare staff and clinicians.^2,12–15^ However, time-motion methods can be time- and labor-intensive given the need for an observer to be physically present in the clinic session to complete an observation. Therefore, in the present study, we adapted prior time study techniques for monitoring clinician time using a virtual observer and used these methods to describe how PCPs spend time when working with the EHR during VHA primary care clinic visits, including in-person, phone, and video visits.

## METHODS

### Design and Conceptual Framework

**Figure 1:**
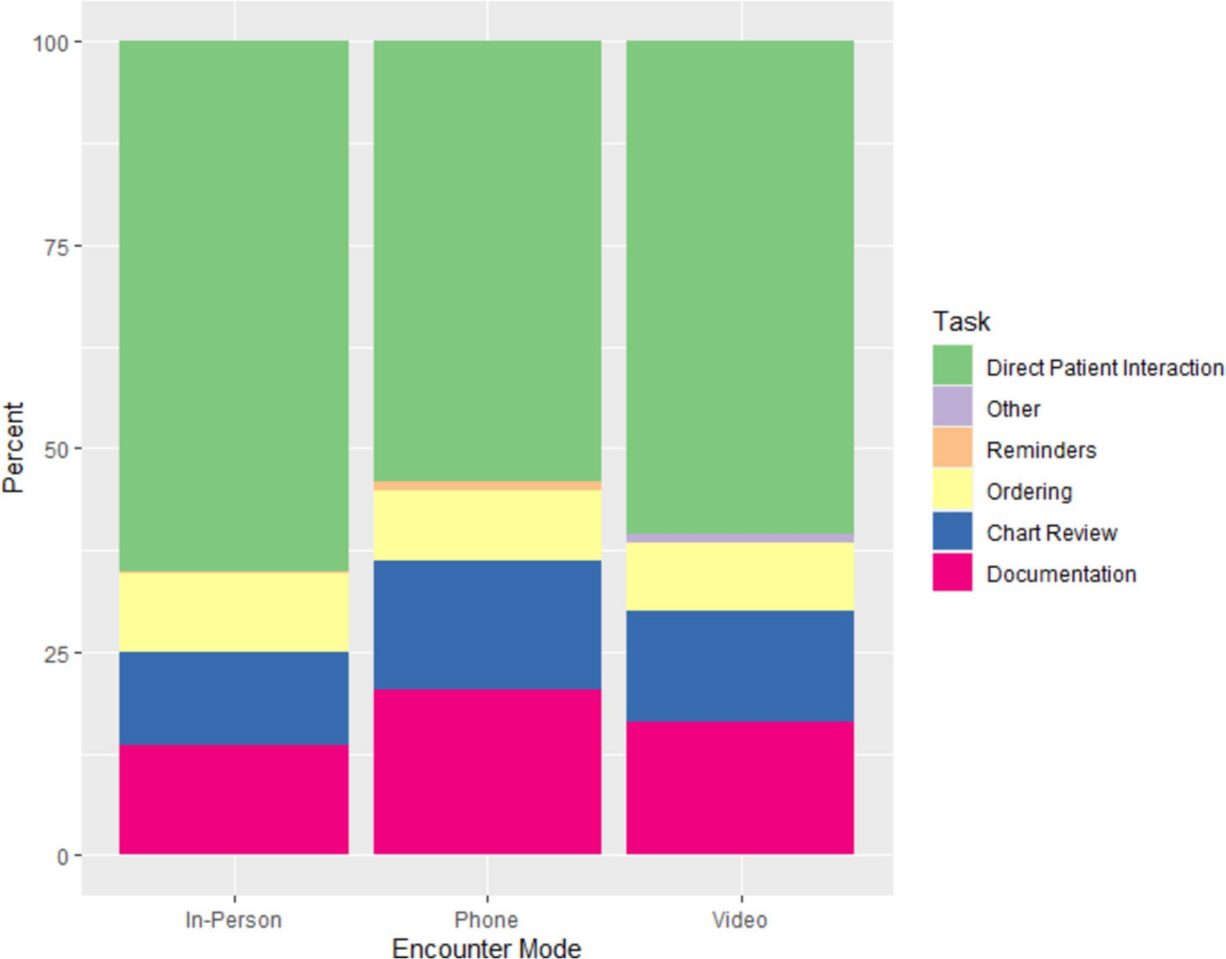
Percent of EHR time spent on each task within a patient visit

We adapted time-motion methods previously used in various healthcare settings to track time allocation among staff and clinicians.^2,15^ Measures of time allocation were mapped within the WorkStudy+ application (Quetech Ltd.), a software for collecting time study data that has been used in other clinical time-motion evaluations.^15^ Details of our data validation for our virtual observation method are described in the Supplement (Supplement Table 1, Figs. 1 and 2).

Due to the COVID-19 pandemic, we developed a protocol for virtual observations using Microsoft Teams screen share. Participating clinicians were scheduled for half-day, 4-h clinic session observations. During the session, an ad hoc video meeting was created, and the clinician shared their screen and audio with the trained observer.

### Study Setting and Participants

A total of 23 PCPs from the three regional VA sites were recruited to participate in the observations from January 2020 through September 2021. Three VA sites from one geographic region (called a Veteran Integrated Service Network (VISN)) participated in the study. The VA Puget Sound Health Care System is a large multicenter healthcare system with two main center sites and eight community clinic locations. The Boise VA Medical Center has one main site and five community clinic locations, and the Portland VA Medical Center has two main campuses and 10 community clinic locations. Exclusions include PCPs from contract clinics, clinicians with less than 25% clinical time, and trainees.

PCPs practice as part of a patient-centered medical home model called the Patient Aligned Care Team (PACT). Each PACT consists of a PCP (which can be a physician, physician assistant, or nurse practitioner), registered nurse, health technician or licensed practicing nurse, and an administrative assistant. One full-time, fully staffed PACT manages a panel of 1200 Veterans. PCPs typically conduct visits in clinic half-day blocks of approximately 4 h of back-to-back scheduled clinic visits. Before the COVID-19 pandemic, in-person routine visits were 30 min, in-person new patient visits were 60 min, and phone visits were 15 min. At the time of this study, the VA was implementing video visits, which were allotted 30 min and could be done in place of an in-person visit. Due to the pandemic, there was increased flexibility and a push to hold more visits via video and phone. Given that many traditionally in-person visits were being done via phone calls, phone visits were also allotted 30 min to accommodate the expanded care needs. All encounters during the participating PCP’s observation window were included unless the Veteran or clinician opted out.

### Clinician Survey

Prior to the scheduled observation, clinicians were anonymously surveyed to capture basic demographics including gender, profession, FTE, years in practice, EHR experience, and self-reported EHR time spent outside of clinic hours (Supplemental Table 2). This survey asked clinician to report their EHR time before/after clinic during the workday, after hours on evenings, and after hours on weekends as separate data points. All sections of the survey were optional for the clinician to complete.

### Data Collection Tools

One observer captured data on an electronic tablet via the WorkStudy+ application (Quetech Ltd.). This software allows for custom task definitions and recording. Descriptions of the contextual models used for time mapping can be found in Supplemental Table 3. Commonly performed tasks within the EHR (i.e., documentation, chart review, order entry, etc.) were coded and mapped to the software interface (Supplemental Fig. 3). Codes were also created for other computer work or interruptions. Any typing, mouse movement, or clicking within the EHR was captured as EHR work. Therefore, all multitasking during a visit, e.g., speaking with or listening to the patient while typing or clicking within the chart or another application, was captured as the time spent in the EHR or other application. If there was a pause for longer than 3 s in screen activity including mouse movement, typing, or scrolling, the observer would record the end of the last task and start a new task of clinical care. For each instance in which the observer records the start of a new task in the software, a timestamp for the start of the new task and the end of the prior task is captured, allowing for cumulative lengths of time to be recorded for individual tasks. Microsoft Teams screen share meeting function was used to virtually observe the interaction with the EHR and listen to the audio from the clinic session. Except for portions of the visit involving a physical examination, the clinician left their camera on for the clinic session, allowing the observer to see clinician gestures and eye movements, and to see when they might leave the room during a clinic session. For all observations in this study, clinicians were using a single clinic room continuously for patient care, and time spent outside of the room was captured as “Transit” in the software.

### Data Analysis

The primary outcome of interest was the percentage of time spent on EHR work within a primary care visit defined as the total number of minutes the clinician spent on the EHR divided by the total observation time within a visit. The time spent within a primary care visit was defined as the time spent between the first and last communication of the patient or clinician within the visit. Secondary outcomes included visit-level percentage of time spent on specific EHR tasks: documentation, records review, ordering, reminders, and data entry within a visit. Secondary outcomes also included between-visit work (i.e., work done in the EHR during the 4-h clinician observation, but before or after the patient visit), defined as the percentage of time spent on tasks between patient encounters and within the clinician’s 4-h observed session. These tasks included documentation, records review, ordering, reminders, alerts, data problems, and coding/billing. We also present descriptive statistics of weekly self-reported hours of EHR work done after hours (evenings and weekends) and outside of clinic sessions during the workday.

For primary and secondary visit-level outcomes, we reported observed mean number of minutes across visits for each of the three visit modalities. We calculated average percent of EHR use using a mixed effects model adjusting for visit mode (phone, in-person, video) and a random effect for clinician to account for multiple visits with the same clinician. For between-visit secondary outcomes, we reported observed mean number of minutes across observation sessions. We calculated the average percent of time on EHR tasks between patient visits using a mixed model adjusting for a random effect for clinician to account for multiple sessions observed with the same clinician. We also calculated descriptive statistics of time on EHR by clinician.

### Ethics

This evaluation was conducted as part of ongoing operational quality improvement project and was designated as a non-research activity by the VHA Office of Primary Care. A memorandum of understanding was signed with the regional union for participating sites.

## RESULTS

### Time-Motion Observations

We observed 23 PCPs for a total of 211 primary care visits (Table 1) in *n*=39 4-h clinic sessions. The average visit length was 23.9 min in-person, 21.2 min by phone, and 29.3 min on video (Table 2). The average proportion of time spent on EHR work during the visit was 35% for in-person visits, 46% for phone visits, and 39% for video (Fig. 1). Of this EHR time, the majority was spent on documentation and chart review (Table 2). The EHR time during visits varied between PCPs, ranging from a low of nearly 20% to over 50% of in-person visit time (Supplemental Fig. 4).

**Table 1:** Demographics of Observed Primary Care Clinicians (*N*=23)

Characteristics	*N* (%)
Age	
31–40	10 (43%)
41–50	5 (22%)
51–60	4 (17%)
61+	4 (17%)
Self-identified gender*	
M	7 (32%)
F	15 (68%)
Years in profession	
<5	3 (13%)
5–10	8 (35%)
>10–20	5 (22%)
>20	7 (30%)
Number of EHRs used**	
1	2 (9%)
2	0 (0%)
3	4 (17%)
4	11 (48%)
5+	6 (26%)
Years at this practice	
<5	14 (61%)
5–10	8 (35%)
>10	1 (4%)
Clinical FTE at this clinic	
0.8–1.0 (full-time)	13 (57%)
<0.8 (part-time)	10 (43%)

^*^N=1 missing for gender

^**^Number of EHRs used includes current and past use

Abbreviations: *M*, male; *F*, female; *FTE*, full-time equivalent

**Table 2:** Observed Time Spent on EHR and Mean Percentages of EHR Use Within Visit (*N*=211 Clinic Visits)

	In-person (*N*=137)		Phone (*N*=65)		Video (*N*=9)	
	Mean time per visit, *m*	Time spent (95% CI), %*	Mean time per visit, *m*	Time spent (95% CI), %*	Mean time per visit, *m*	Time spent (95% CI), %*
Total visit time	23.9	-	21.2	-	29.3	-
Total EHR	8.4	35% (32–38%)	10.0	46% (42–50%)	11.6	39% (31–47%)
Documentation	3.3	14% (11–16%)	4.7	20% (17–24%)	5.0	16% (9–24%)
Chart review	2.8	11% (9–14%)	3.2	16% (13–19%)	4.0	14% (8–19%)
Ordering	2.3	10% (8–12%)	1.8	9% (6–11%)	2.5	8% (3–14%)
Reminders	0.1	0.2% (0–0.7%)	0.3	1% (0.5–1.7%)	0	0.1% (0%,1.7%)

This table excludes “Data Entry/Problem List” since there were fewer than 5 encounters with non-zero values. Documentation refers to actions in which the clinician is typing, clicking, scrolling, or dictating within the visit’s clinical note. Chart review, called “Results Review” in the WorkStudy+ application (Supplemental Table 3), includes actions within a patient’s chart such as reviewing other clinical notes, test results, medications, and problem lists. Ordering includes actions to create, edit, or sign orders. Reminders include actions to complete a dialog within the EHR for health screening or preventative health items

^*^Adjusted percentages are from the modelled visit-level percentages

During the observed 4-h clinic sessions, PCPs spent 54 min on average performing administrative tasks within the EHR between patient visits (Table 3). Of this between-visit time, 44% was spent on documentation, 14% on chart review, and 14% on placing orders (Table 3).

**Table 3:** Observed Time Spent on EHR Between Patient Visits (*N*=39 4-h Clinician Observation Sessions)*

	Mean time per observed half-day session, *m*	Time spent (95% CI), %**
Total between-visit time	69.5	-
Total EHR	54.0	75% (69–80%)
Documentation	34.0	44% (39–49%)
Chart review	10.0	14% (10–18%)
Ordering	8.7	14% (10–18%)
Alert inbox	1.1	1.4% (0.2–2.5%)

^*^Excludes “Data Entry/Problem List,” “Reminders,” and “Coding/Billing” since fewer than 8 sessions with non-zero values

^**^Adjusted percentages are from the modelled visit-level percentages

### Clinician Survey

All but one PCP observed (*n*=22) completed the portion of the clinician survey regarding time spent on administrative work related to patient care outside of clinic sessions and after hours (Table 4). PCPs reported spending an additional 0.3–4 h (average = 2.4 (SD=1.00)) on administrative work per workday outside of clinic sessions. More than half (59%) reported spending evening or weekend hours completing clinical work.

**Table 4:** Self-reported Time Spent on Administrative Work Related to Patient Care Outside of Clinic Sessions (*N*=22 Clinicians) (One PCP Did Not Fill Out This Portion of the Clinician Survey)

	Average (SD)	Median	Range
No. of hours per workday outside of clinic sessions	2.40 (1.00)	2.2	0.33–4.0
Clinician working weekends or evenings, *N* (%) (*n*=13, 59%)			
No. of days per week working evenings/weekend	3.62 (2.06)	3.0	1.0–7.0
No. of hours per weekday evening	1.37 (0.89)	1.0	0–3.0
No. of hours per weekend	2.29 (2.28)	1.3	0–8

## DISCUSSION

Our results show that PCPs at the VHA spend between one-third and one-half of each patient visit interfacing with the EHR. Additionally, between patient visits during clinic sessions, PCPs spend 75% of their time working in the EHR. In total, this represents over 2 h per half-day clinic session spent on EHR tasks. Time spent on EHR work, while necessary, may negatively impact communication with patients,^7^ and contributes to burnout among PCPs.^5^ Our findings indicate wide clinician variability in time spent on EHR tasks, which may reflect individual practice patterns, in addition to other factors such as clinical experience, panel size, patient complexity, and team- or clinic-level support.^16^ Despite efforts at VHA to reduce the burden of the EHR,^9^ the burden remains high and is likely to persist without broader systemic changes.

This is the first observational study within the VHA documenting how clinicians spend their time directly caring for patients. To date, other major studies of primary care EHR time done outside the VHA have shown that clinicians spend 4.5 h, or nearly half of total time, interfacing with the EHR during their workday and 1–2 h in the EHR after hours each day.^2,15^ We found that time spent on EHR work among VHA PCPs is similar to that at non-VHA clinics. In our study, most of this time is spent on documentation and chart review. Addressing documentation burden through the use of artificial intelligence (AI)-driven ambient dictation shows promise for reducing workload.^17–19^ Additionally, although in its infancy, AI-enhanced EHRs may not only support clinicians only in completing chart review more efficiency, but also provide real-time clinical decision support to improve patient care.^20–22^

To our knowledge, our study was the first to employ a virtual method for observing clinician time. While initially developed for practical reasons due to the COVID-19 pandemic, we found the virtual method had the added benefit of increased scheduling flexibility and remote data capture. This virtual method of task observation may also prove useful among harder to observe clinical sessions such as smaller clinic rooms and rooms with patients requiring isolation precautions. It also allows for greater efficiency of data capture across distant sites. Additionally, this is the first evaluation to our knowledge to include observation of virtual visits (i.e., phone, video). The pandemic increased virtual primary care visits,^11^ leading to a sufficient volume of visits for us to make observations on differences in the relative proportion of clinician tasks occurring in different visit modalities. We found that between one-third and nearly one-half of observed visit time is spent on work in the EHR. EHR time was greatest for phone visits, during which the patient is unable to directly see the clinician. We suspect that in-person and video visits generate more between-visit work in documentation, chart review, and order entry because less EHR work is accomplished during the visit. Phone visits are also noticeably shorter than the other visit types, which is likely due to lower complexity visits being scheduled for phone rather than in-person or video,^23^ and lack of physical examination time.

While we did not observe time spent outside of the half-day clinic session, self-reported surveys indicated variability between clinicians. On average, clinicians spent 2.4 h per workday completing administrative work related to patient care outside of clinic sessions. Additionally, over 50% of the observed PCPs spent additional time completing administrative tasks after hours and on weekends. Unfortunately, the time spent on work outside of clinic sessions among PCPs at the VHA is no better than those at non-VHA clinics.^2^ This work may have implications for addressing workload, administrative-clinical balance, and patient-clinician relationships, all of which impact burnout.

Lastly, VHA has begun undertaking an EHR transformation in migrating from CPRS to a commercial product. EHR transitions are a notoriously difficult time for healthcare systems. Given the required training and time to learn the new systems, there can be temporary disruptions, increased staff turnover, and cognitive workload burden to clinical staff.^24,25^ The VHA’s EHR transition may impact EHR time during and outside of patient visits. Our novel virtual method for conducting a time-motion study provides a useful baseline for future work around administrative workload related to EHR transition.

### Limitations

There are several limitations to our reported work. Generalizability is limited as all PCPs were from one geographic region and the study’s sample size was 39 4-h clinic observations among 23 PCPs. Therefore, we were unable to analyze differences in EHR behavior according to clinician demographic characteristics such as years in practice, professional discipline, and gender. Additionally, we did not analyze the effect of different patient medical comorbidities, which may have a significant impact on the content of a visit and the needs for interaction with the EHR.

The time period for this study from January 2020 through September 2021 coincided with the onset of the COVID-19 pandemic. With a dramatic increase in virtual visits and decrease in in-person visits during this time,^11^ the reasons for visits scheduled in these modalities may differ substantially from visits in years before and after the onset of the pandemic. Additionally, because video visits were a relatively new modality during this period, lack of patient and clinician experience with video visits may have impacted clinician EHR behaviors.

We only captured workflow during a clinic session and did not directly measure the clinical and EHR-related administrative work performed outside of the workday. While possible to measure timestamp data for VA EHR tasks after hours, this was beyond the scope of our work. Instead, we relied on self-reported estimates, which are shown to be less reliable.^12^ Furthermore, due to the virtual nature of the observation, only one screen could be seen at a time, which limited the ability to fully capture details of non-EHR tasks and likely led to underreporting of interruptions and overall computer time during visits. The method was also limited in its ability to capture work-related tasks that were not performed in-person and outside the EHR.

PCPs may interact differently with the EHR while under direct observation (the Hawthorne effect), making it difficult to know to what extent the clinician’s behavior during the observations reflects their usual practice. Finally, our study did not attempt to capture data on the quality of care rendered by PCPs or patient satisfaction. Therefore, we cannot assess whether there was an association between the amount of time spent on different EHR tasks and these outcomes.

## CONCLUSIONS

To our knowledge, this work is the first to use a virtual adaptation of a previously validated time-motion method^15^ and is the first observation of both in-person and virtual care visits among PCPs at the VHA. We found that PCPs spend a significant proportion of the clinic visit interfacing with the EHR. Importantly, when compared to phone or virtual visits, less time was spent in the EHR during in-person visits. This suggests that the EHR may be a barrier to communication with the patient when present in the room — which may translate to spending significant hours outside of clinic sessions each day on administrative tasks. Based on self-report, it does not appear that time spent working within the EHR after hours has improved. This work is essential to understanding PCP efficiency and workload at the VHA and can be used for primary care planning and policy.

## Supplementary Information

Below is the link to the electronic supplementary material.

Supplementary file1 (DOCX 169 KB)
