# Supplementary material for: Electronic Health Record Time Allocation Among Primary Care Clinicians at the Veterans Health Administration Using Virtual Observations
Source: J Gen Intern Med. 2025 Jan 6;40(9):2087–93. doi: 10.1007/s11606-024-09328-y (PMC12325830; doi:10.1007/s11606-024-09328-y)
Supplement: Supplementary file 1 — Supplementary file1 (DOCX 169 KB) [file 11606_2024_9328_MOESM1_ESM.docx]

**Supplement**

Description of Time-Motion Virtual Observation Technique Validation
To validate time-motion observations, the lead author (SD) observed video recordings of the computer screen during 10 scenarios, which were a mixture of mock in-person, phone, and video visits, as well as between visit work. The lead author recorded time allocation using the WorkStudy+ application interface on an Apple iPad. These observations were considered the gold standard. One observer (SK) was trained on the application and validation was performed on their observation of the same mock scenarios. The one trained observer conducted all study observations.

The goal of the validation analysis was to determine whether all primary and secondary outcomes derived from the WorkStudy+ application were reliable and reproducible. To determine if outcomes were reliable, we compared outcomes derived from the rater’s observations to a “gold standard” on 10 independent test visits. We calculated the inter-rater correlation coefficient, defined to be the propensity for the rater to record a value similar to the gold standard value, chance agreement having been removed, and plotted the Bland Altman plot. To determine if outcomes were reproducible, we compared outcomes derived from the only rater’s first observations to the same rater’s second observations on the same 10 test visits. We calculated an intra-rater reliability coefficient, defined to be the propensity for the rater to record a value on their second observation similar to the value from their first observation on the same test data, chance agreement having been removed.
**Results of Validation Study**

For the validation analyses using mock patient visits, all observations for reliability fell within two standard deviations of the average difference between the rater and the gold standard. This indicated agreement and satisfied reliability for the outcomes considered (Supplemental Figure 1). The inter-rater correlation coefficient for the primary outcome was 0.931 which also indicates good reliability. All but one observation for reproducibility fell within two standard deviations of the average difference between the rater’s first try and the rater’s second try, indicating agreement and satisfactory reproducibility for the outcomes considered (Supplemental Figure 2).

**Supplemental Table 1: Reliability Results for the Primary Outcome (Proportion of Time on the EHRM)**

| **Video Session** | **Rater 1st Attempt** | **Gold Standard** | **Rater 2nd Attempt** |
| --- | --- | --- | --- |
| **Video #1** | 0.427 | 0.438 | 0.403 |
| **Video #2** | 0.435 | 0.520 | 0.477 |
| **Video #3** | N/A* | N/A* | N/A* |
| **Video #4** | 0.283 | 0.277 | 0.254 |
| **Video #5** | 0.489 | 0.488 | 0.432 |
| **Video #6** | 0.595 | 0.572 | 0.573 |
| **Video #7** | N/A* | N/A* | N/A* |
| **Video #8** | 0.371 | 0.381 | 0.313 |
| **Video #9** | 0.555 | 0.638 | 0.492 |
| **Video #10** | 0.379 | 0.382 | 0.339 |

* N/A if there is no patient visit in the video.

**Supplemental Figure 1. Bland-Altman Plot for Primary Outcome: Proportion of Time Spent on EHRM in a Patient Visit for Validating Reliability***


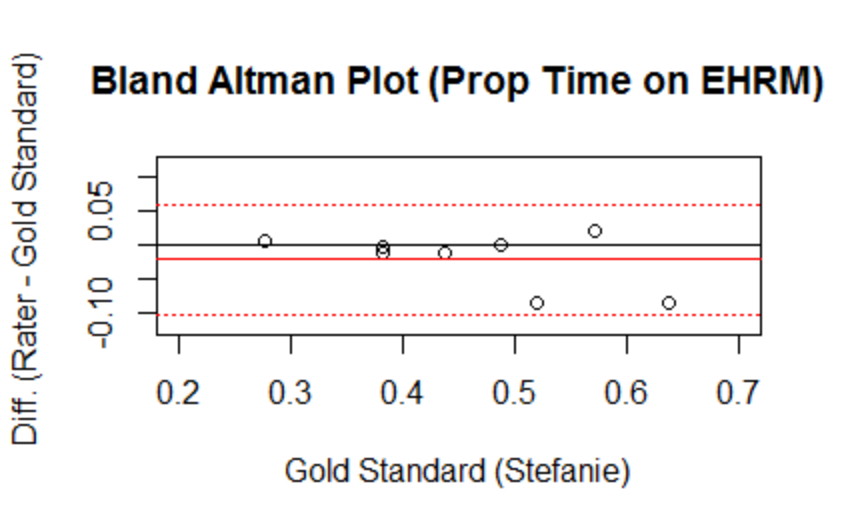


*Solid black horizontal line is at 0; red solid line at the mean of the differences between the rate and the gold standard; red dotted lines at 2 standard deviations above and below the mean difference.

**Supplemental Figure 2. Bland-Altman Plot for Primary Outcome: Proportion of Time Spent on EHRM in a Patient Visit for Validating Reproducibility***
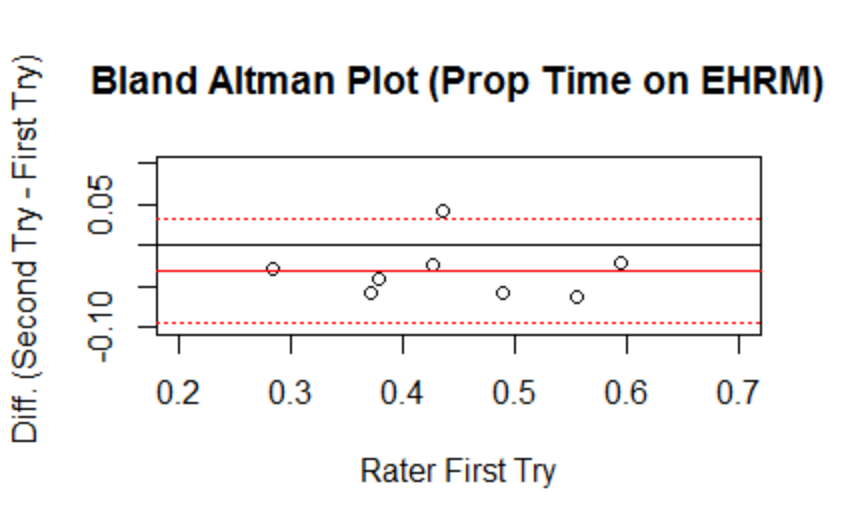


*Solid black horizontal line is at 0; red solid line at the mean of the differences between the rate and the gold standard; red dotted lines at 2 standard deviations above and below the mean difference.

**Supplemental Table 2. Clinician Demographic Survey**

| **Age** | 20-30 years  31-40 years  41-50 years  51-60 years  61+ years |
| --- | --- |
| **Gender Identity** | M  / F / non-binary / decline |
| **Years in medical profession (since first non-training licensure)** | _________  years |
| **How many different EHRs have you used?** | ______ EHRs |
| **Years at THIS practice** | _______ years |
| **Clinical FTE in this clinic?** | ______ FTE (Must be >/= 0.25) |
| **Patient clinical hours (face to face/ tele/video) per week** | ______ hours/week |
| **Any portion FTE to non-clinical activities such as Education, Research, Practice Management** | Y / N, if yes, FTE portion? |
| **On average, how many minutes/hours per day spent doing clinic administrative tasks before/after clinic sessions AT WORK?** | _________ hours/minutes per day. |
| **Do you do clinic administrative work evenings and weekends? If yes, how many days per week?** | Y/ N /Other –  ________ days per week. |
| **On average, how many minutes/hours spend on clinic administrative tasks AT HOME in evenings?**  **On weekends?** | _________ evening hours/mins.  _________ weekend hours/mins. |
| **Can you tell me the break down for today’s visits?** | ______tele/ _____FTF/ _____VVC |
| **Can you tell me what goes into your/your patients decision to select the visit type (i.e. face-to-face vs. VVC vs. tele)?** |  |

**Abbreviations:** VVC – VA Video Connect, VHA’s video visit platform.

**Supplemental Figure 3. Workstudy+ Application Interface Map for Observation and Coding of Tasks**

| **Tasks – layer I** | | **Context - II** | | **Unobserved Time** |  |
| --- | --- | --- | --- | --- | --- |
| Clinical Care | Transit | EHR | Internet, Software | Closed to Observation | **Encounter Start** |
| Documentation | Personal | EHR Inbox  “View Alerts” | Phone | Observation Technical Issue | **Encounter End** |
| Data entry/ Reminders | Communication | Email | Paper | Clinician Out of Room | **Delay** |
| Ordering | Crashed Tech | Teams | Video | **With Whom -IV** | Interruption |
| Results Review | Other |  | Tech Other | Patient | Logging On |
|  |  | **VISIT TIME - V** | | Staff | Other |
|  |  | During visit | Between Visits | Other | **On Break** |
| **End Session** |  |  |  |  | **Comments** |

Example of home screen layout within the application. Each box represents a button that can be clicked through to reach additional modifiers. Multiple contextual layers can be applied to each timed recording.

**Supplemental Table 3. Coding of EHR Tasks within the WorkStudy+ Application**

| **CONTEXT I** | **WHAT / Task** |
| --- | --- |
| **Clinical Care**  ***Any care that directly involves a patient*** | Scheduled visits with patient interaction  Face to face, Phone visit, video visit interaction with patient  Examples:  Interviewing patient or family  Making a management plan  Physical exam  Performing procedure  Discussing facts with patient  Reviewing instructions with patient |
| **Documentation** | ACTION identifies interaction with record or source. May include: writing, typing, scrolling, clicking, recording/dictating.  Can usually tell by clinician in chart typing quickly    Examples:  Typing during patient encounter when talking with patient, this is Documentation, Exam, Patient, EHR  Typing in note or letter field after patient has left the clinic room or encounter has ended or after clinic hours |
| **Data Entry/Reminders** | Asking a patient questions related to health screening or preventative health, typing answer into alert/clinical reminder  Trigger word “clinical reminder” or “health factor” or “problem list”  This is intended to capture fill of mandated data fields (ex. Positive Depression, Columbia Screen, Pneumonia Vaccine, Colorectal cancer screening)  Adding a problem/diagnosis code to the problem list |
| **Results Review** | ACTION of review results from a SOURCE (Mode like EHR, paper, etc)  If results are checked in front of a patient this is coded: Results Review, Exam Room, Patient    Example:  Scanning lists of obtained results  Searching for notes from consult/specialist  Reviewing paper or electronic faxes (usually PDFs to review/sign)  Asking a nurse or assistant to check  Can be “checking JLV”  Sometimes a “to do” list  Reviewing chart before entering room with patient    Types of Results  Lab, procedure result, biopsy result  Imaging result  Result from consult, note or letter |
| **Order Entry** | Requires ACTION, typing or speaking to someone (EHR or phone)  CUE name of medication, name of lab, name of consult, “refer you to X” “order by mail or window/pick up”    Example of order types  Labs  Medication  Imaging  Consult  Vaccines |
| **Medication Orders** | Typing/searching for medication order in EHR  Signing medication order  Directing staff member to write script or to refill (refill staff delegate)  Calling pharmacy to refill, ask about availability, dosage, recommendation  IMing pharmacy staff to ask about refill, availability, dosage, advice |
| **Other Orders** | Ordering lab, pathology, microbiology test  Ordering imaging test  Ordering consults  Ordering medical equipment or device  Calling lab to tell them about a rush order, add on, or special order |
| **Transit** | Relocating place to place  No interactions |
| **Personal** | Not work related  Examples:  Bathroom break  Eating (can be at desk, multitasking)  Making a personal call or text |
| **Communication** | Outside of visit, before or after  Can usually tell by clinician in chart or email and typing quickly  Letters or emails to other clinicians (these are recordings of clinical work)  Calling pharmacy to refill, ask about availability, dosage, recommendation  IMing pharmacy staff to ask about refill, availability, dosage, advice  Calling or messaging other staff or consultants  Answering a page or call  “Huddles” with staff to prepare for day. Huddles may include some workflow discussion, this still counts as pre-work  At start of day “running the list” to review patients before seeing them in clinic |
| **Other** | Closed to observation  Education or meeting  Performing non-direct clinical administrative tasks (academic, holding administrative meeting) |
| **CONTEXT II** | **HOW / Mode of Task** |
| **EHR** | Software sourcing patient information (i.e. CPRS or Cerner)  Clinical management system on computer or tablet  Examples  EHR “Inbox” or manner of task triage, or internal communication “view alerts” related to patient care |
| **EHR Inbox** |  |
| **Email** | Email messaging NOT embedded in EHR  Clinician may have two or three |
| **Microsoft Teams/Messaging** | Direct messaging NOT embedded in EHR |
| **Internet/Software** | Reviewing or searching for records on web interface not imbedded in EHR (Joint Legacy Viewer, “JLV”, VISTA)  VISTA imaging/PACs systems not embedded EHR  MyHealtheVet patient email  Other computer management systems NOT embedded in EHR (ex. DST, Schedule (VISTA Visual Aid)) |
| **Phone** | Personal or office device  Landline or Mobile  Vocera |
| **Video** | Using computer or ipad  Visit with patient or family  Keyword “VA Video Connect” or “VVC” |
| **Paper** | Fax print outs  Paper to do list  Paper print outs  Paper schedules  Whiteboards |
| **Other Tech** | Example - Dictation/ Dragon/ talking to scribe |
| **CONTEXT III** | **WHERE / Location*** |
| **Exam Room** | Clinical work done in room with patient  Procedure room, even if preparing for procedure and patient not there yet |
| **Work Station** | Desk where they do their work  “Team rooms”  Look for personal artifacts (bags, coats, family pictures, lunch)  Using a tablet, but at the same counter or desk each time |
| **Other** | Hallway, reception, nurses station, conference room |
| **CONTEXT IV** | **WHO*** |
| **Patient** | Patient or family |
| **Staff** | Clinical or Office |
| **Other** | Medical student or observers |

*WHO and WHERE were planned contextual modifiers for in-person observations. Due to conducting the observations virtually, these two modifiers were not used during the study period. We show them here for completeness and those considering in-person observations.

**Supplemental Figure 4.** Clinician level variability in proportion of time spent on EHR during an in-person visit*


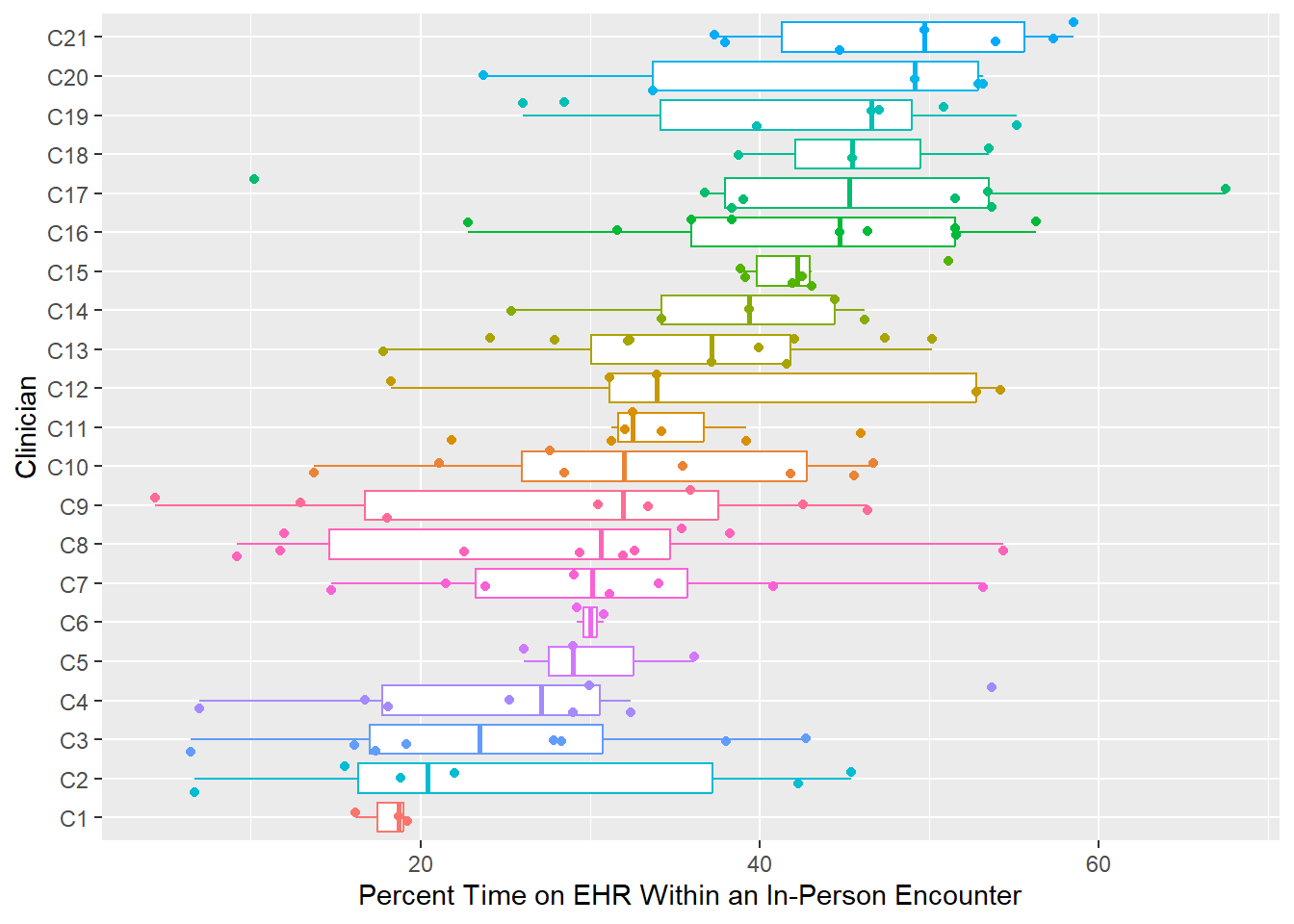


*Dots represent observed EHR percentages for each encounter; median, quartiles, and range are depicted in this plot.

N = 21 as one clinician had no in-person encounters during their observed session.
